# Epulis: à propos d'un cas

**DOI:** 10.11604/pamj.2014.17.19.2997

**Published:** 2014-01-17

**Authors:** Amal Akazane, Badreddine Hassam

**Affiliations:** 1 Service de Dermatologie/Vénérologie CHU Ibn Sina, Faculté de Médecine et de Pharmacie Med V Souissi, Rabat, Maroc

**Keywords:** Epulis, dent, gencive, Epulis, tooth, gum

## Image en medicine

L'épulis est une tumeur bénigne de la gencive qui n'a aucun potentiel dégénératif. Le nom vient du grec, épi = extérieur et oulon = gencive. La tumeur apparait généralement comme une excroissance suite à une irritation locale chronique, ou des perturbations hormonales ce qui explique sa relative prédilection chez la femme en période dactivité génitale et la femme enceinte. Cliniquement elle se présente sous forme de masse charnue rouge foncé très vascularisée, saignant facilement au contact, circonscrite sessile ou pédiculée, Histologiquement, l'épulis distingue différentes formes : Epulis inflammatoire, à cellules géantes, fibreuse, gravidique, congénitale, granulomateuse, le traitement consiste en l'éxerèse chirurgical. Nous rapportons le cas d'une patiente de 46 ans qui consulte pour une tumeur de la gencive inférieure évoluant depuis 10 mois soit 6 mois du post partum ; l'interrogatoire n'a révélé aucun antécédent particulier. L'examen clinique objective au niveau de la gencive inférieure une masse indolore, exophitique, en forme de framboise, ferme , sessile saignant au contact, avec un mauvais état bucco-dentaire le reste de l'examen clinique était normal ,une radiographie panoramique réalisée était sans particularité .les données cliniques et radiologiques étaient très évocateur du diagnostic d'épulis. Ainsi l'exérèse chirurgical avec examen anatomopathologique a confirmé le diagnostic en montrant un tissu conjonctif, riche en bandes de collagène, peu cellulaire, richement vascularisé, avec des vaisseaux hyperplasiques, à paroi épaissie. La cicatrisation était bonne et la patiente était suivie par la suite pour des soins dentaires.

**Figure 1:**
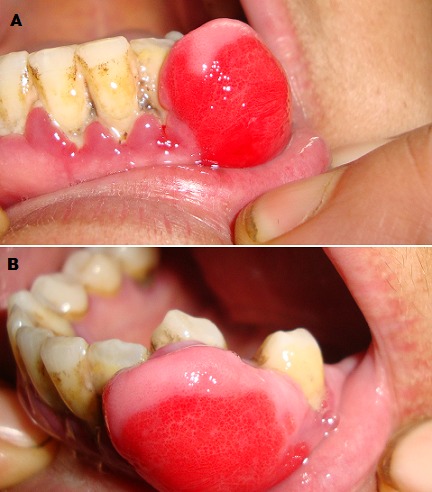
A) Tumeur ferme de la gencive inférieure; B) Vue de profil de la lésion

